# Supplementary figures and images for: Differential viral RNA methylation contributes to pathogen blocking in Wolbachia-colonized arthropods
Source: PLoS Pathog. 2022 Mar 16;18(3):e1010393. doi: 10.1371/journal.ppat.1010393 (PMC8959158; doi:10.1371/journal.ppat.1010393)

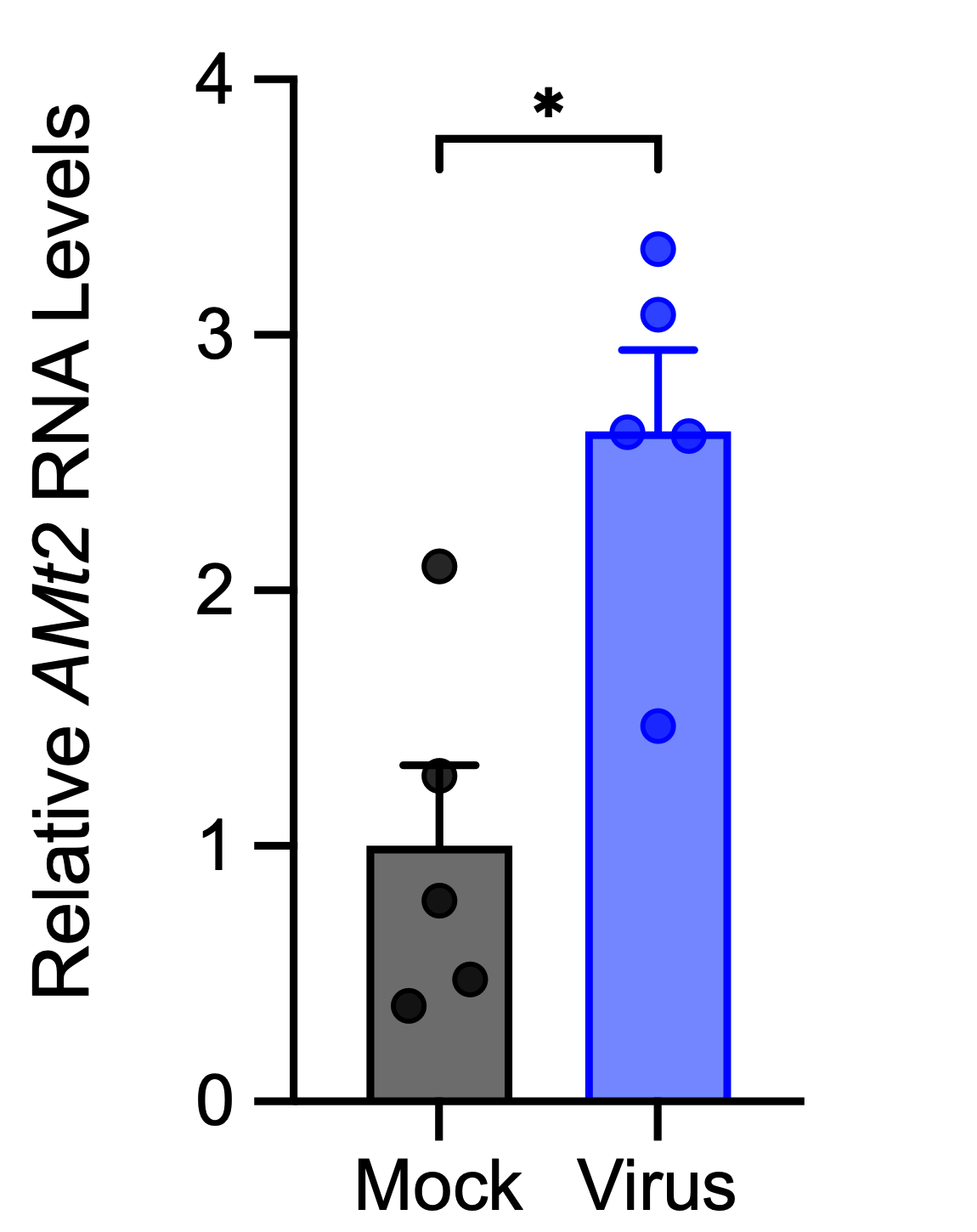

Supplement: S1 Fig — Relative AMt2 expression in the presence (Virus) and absence (Mock) of Sindbis virus in Aedes albopictus cells. Quantitative RT-PCR was used to measure relative mRNA levels of AMt2 in Aedes albopictus cells following virus infection initiated with an MOI of 10. Unpaired Mann Whitney U-test. Error bars represent standard error of mean (SEM) of independent biological replicates. Primer details are available in S1 Table. *P<0.05. (TIF) [file ppat.1010393.s001.tif]

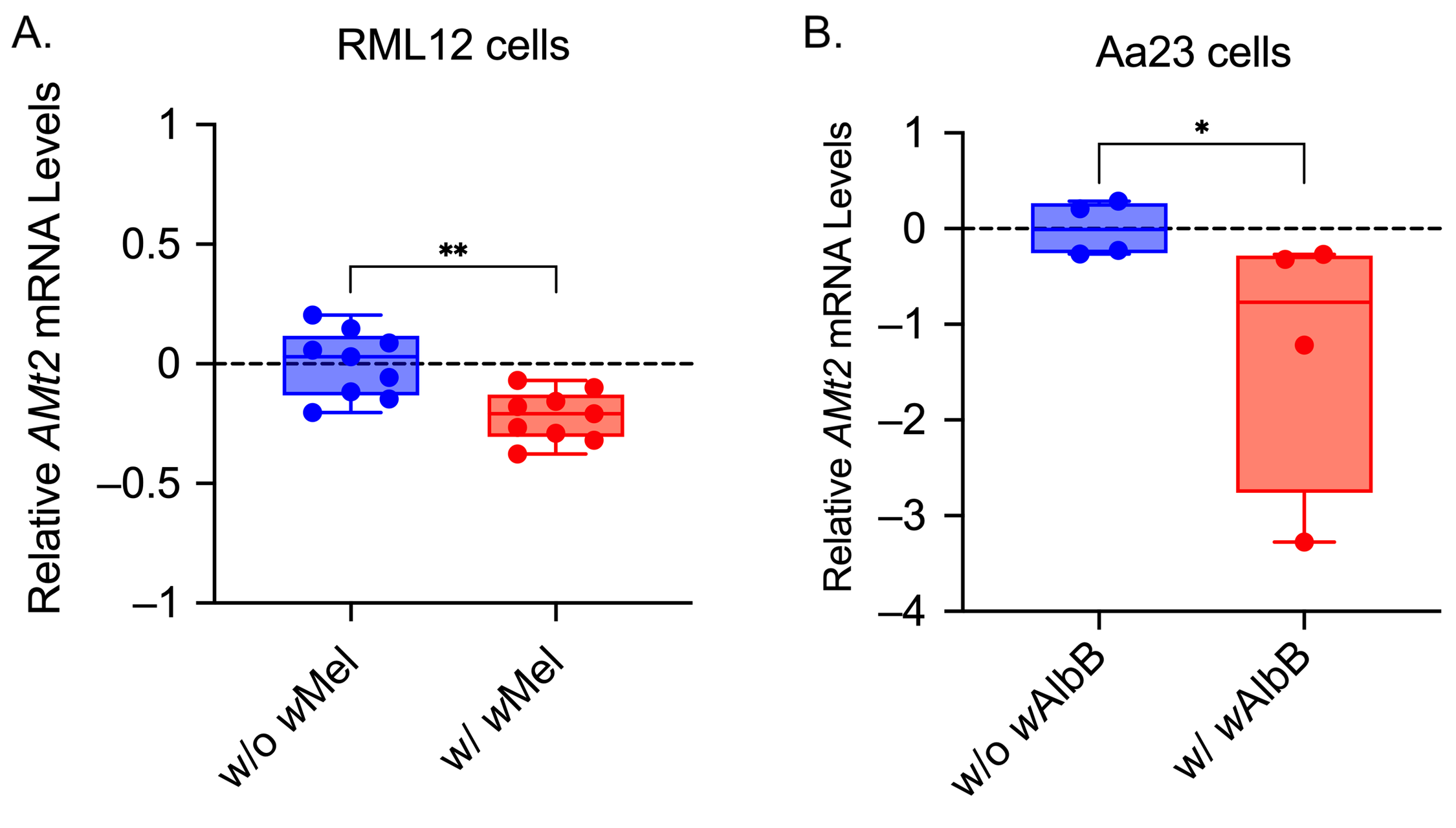

Supplement: S2 Fig — Relative AMt2 expression in the presence (w/ Wolb) and absence (w/o Wolb) of Wolbachia in Aedes albopictus cells. Quantitative RT-PCR was used to measure relative mRNA levels of AMt2 in Aedes albopictus cells colonized with (A) wMel strain of Wolbachia (RML12) and (B) wAlbB strain of Wolbachia (Aa23). Unpaired Mann Whitney U-tests on log-transformed values. Error bars represent standard error of mean (SEM) of independent experimental replicates. Primer details are available in S1 Table. **P < 0.01, *P < 0.05. (TIF) [file ppat.1010393.s002.tif]

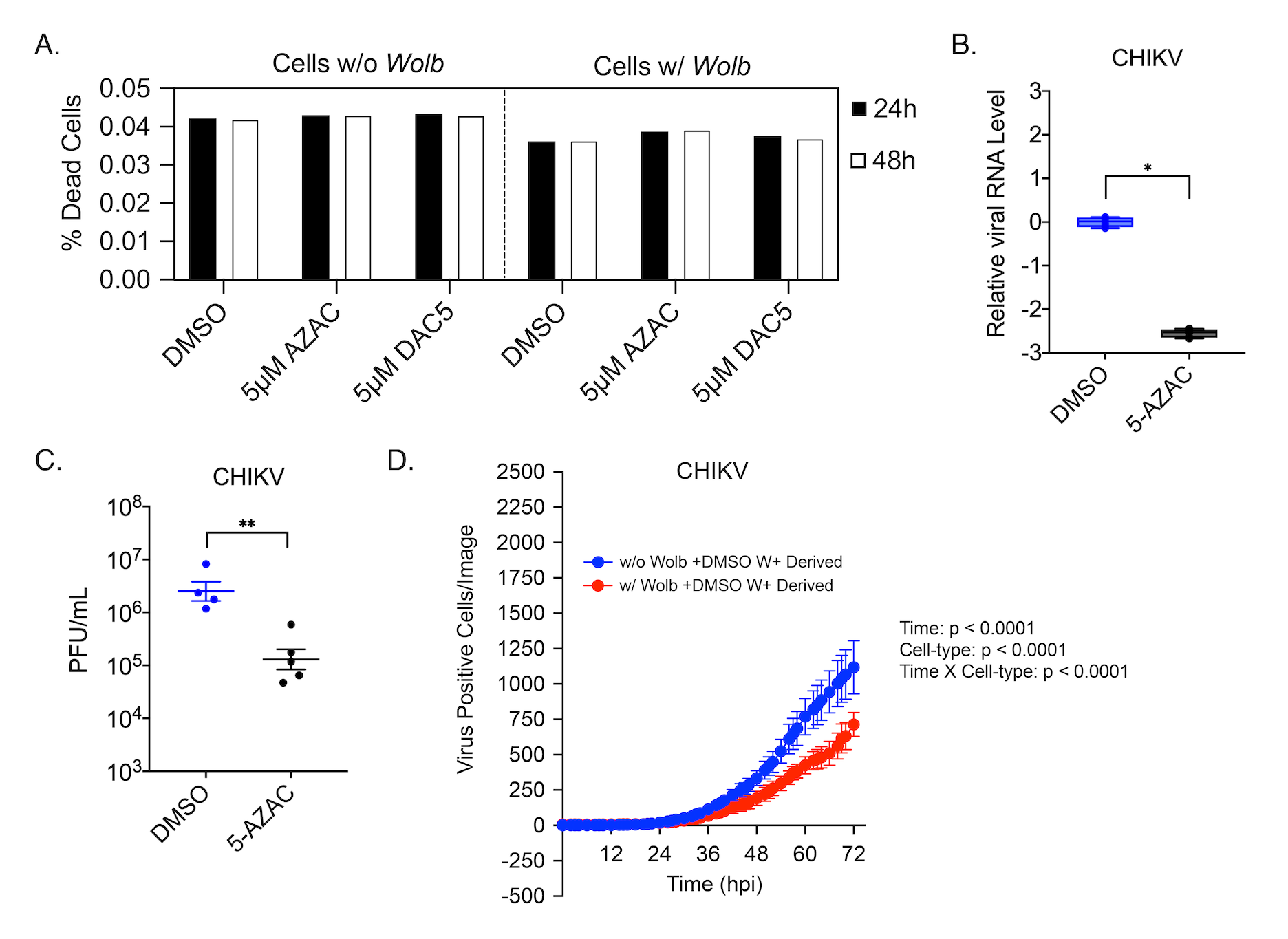

Supplement: S3 Fig — Inhibition of mosquito DNMT2 in Wolbachia-free Aedes albopictus derived C7/10 cells was carried out using MTase inhibitors 5-Azacytidine (5-AZAC), or 5-Deoxyazacytidine (DAC5). Dimethyl-sulfoxide (DMSO) was used as the negative control. In each case, cells were pretreated with 5 μM inhibitors overnight prior to infections with CHIKV-mKate virus at MOI of 10. Cell lysates and supernatants were harvested at 48 hours post infection to quantify cellular viral RNA levels and infectious titer, respectively. (A) Effect of MTase inhibitors on cell death was assayed using Cytotox Reagent and the IncuCyte live-cell imaging platform. Y-axes represent the percentage of stained dead cells quantified under each treatment condition for two experimental replicates. (B) Levels of CHIKV RNA in mosquito cells treated with MTase inhibitor 5-AZAC were determined using quantitative RT-PCR. Unpaired two-tailed t-test with Welch’s correction, CHIKV viral RNA: p < 0.0001, t = 35.30, df = 6.001. (C) Infectious CHIKV titers produced from mosquito cells treated with MTase inhibitor 5-AZAC were determined using plaque assays on BHK-21 cells. Unpaired two-tailed t-test with Welch’s correction, CHIKV titer: p = 0.0019, t = 4.864 df = 6.940 (D) Growth of W+ virus in naïve C7/10 cells without (blue circles) and with (red circles) Wolbachia (strain wStri) pretreated with DMSO control. Two-way ANOVA with Tukey’s post hoc test for multivariate comparisons. Error bars represent standard error of mean (SEM) of independent experimental replicates (n = 3). Time: < 0.0001, Cell-type: p < 0.0001, Time X Cell-type: p < 0.0001. (TIF) [file ppat.1010393.s003.tif]

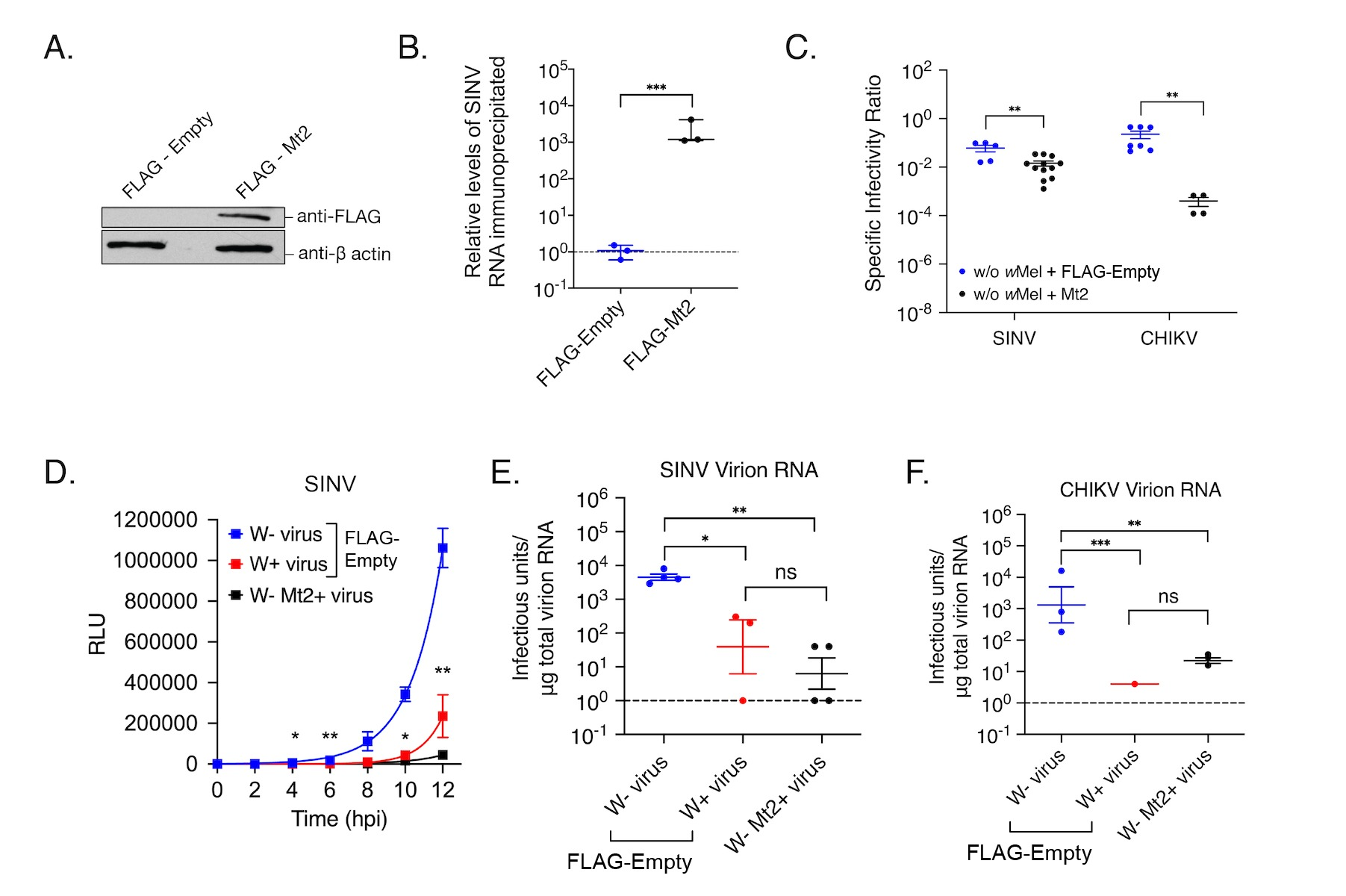

Supplement: S4 Fig — (A) Western Blot of fly DNMT2 in Wolbachia-free Drosophila melanogaster derived JW18 cells transfected with expression vector constructs with (FLAG-Mt2) or without (FLAG-empty) Mt2. Cytoplasmic lysates of cells were collected 72 hours post transfection and probed with anti-FLAG and anti-β actin antibodies. (B) Relative levels of viral RNA recovered following AZA-IP of Mt2 in fly cells was quantified using qRT-PCR. JW18 fly cells without Wolbachia were transfected with expression vectors FLAG-empty or FLAG-Mt2 for 72 hours prior to infection with SINV at MOI of 10. Cells were treated for approximately 18h with 5 μM 5-Azacytidine to covalently trap Mt2 with its target cellular RNA prior to RNA immunoprecipitation using anti-FLAG antibody. One-sample two-tailed t-test performed on log-transformed values, p = 0.001, t = 4.462, df = 11. (C) Specific Infectivity Ratios of progeny viruses derived from Drosophila melanogaster cells colonized with native Wolbachia strain wMel. Fly cells without Wolbachia were transfected with expression vectors FLAG-empty (w/o Wolb) or FLAG-Mt2 (w/o Wolb + Mt2) for 48 hours prior to infection with SINV-nLuc or CHIKV (MOI = 10). Specific Infectivity Ratios of the progeny viruses generated 96 hours post infection were calculated as before. Unpaired two-tailed t-test with Welch’s correction, SINV, p = 0.0045, t = 3.698, df = 9.458, CHIKV, p < 0.0001, t = 9.608, df = 6.926. (D) Progeny SINV-nLuc derived from fly cells with (W+ virus), without (W- virus) Wolbachia or overexpressing Mt2 (W- Mt2+ virus) were subsequently used to synchronously infect naïve BHK-21 cells at equivalent MOIs of 5 particles/cell. Cell lysates were collected at indicated times post infection and luciferase activity (RLU), was used as a proxy for viral replication. Two-way ANOVA Tukey’s multiple comparisons tests, Time: p < 0.0001, Wolbachia/AMt2: p = 0.0002, Time X Wolbachia/AMt2: p < 0.0001. (E and F) Overexpression of Drosophila DNMT2 ortholog reduces infectivity of pro [file ppat.1010393.s004.tif]

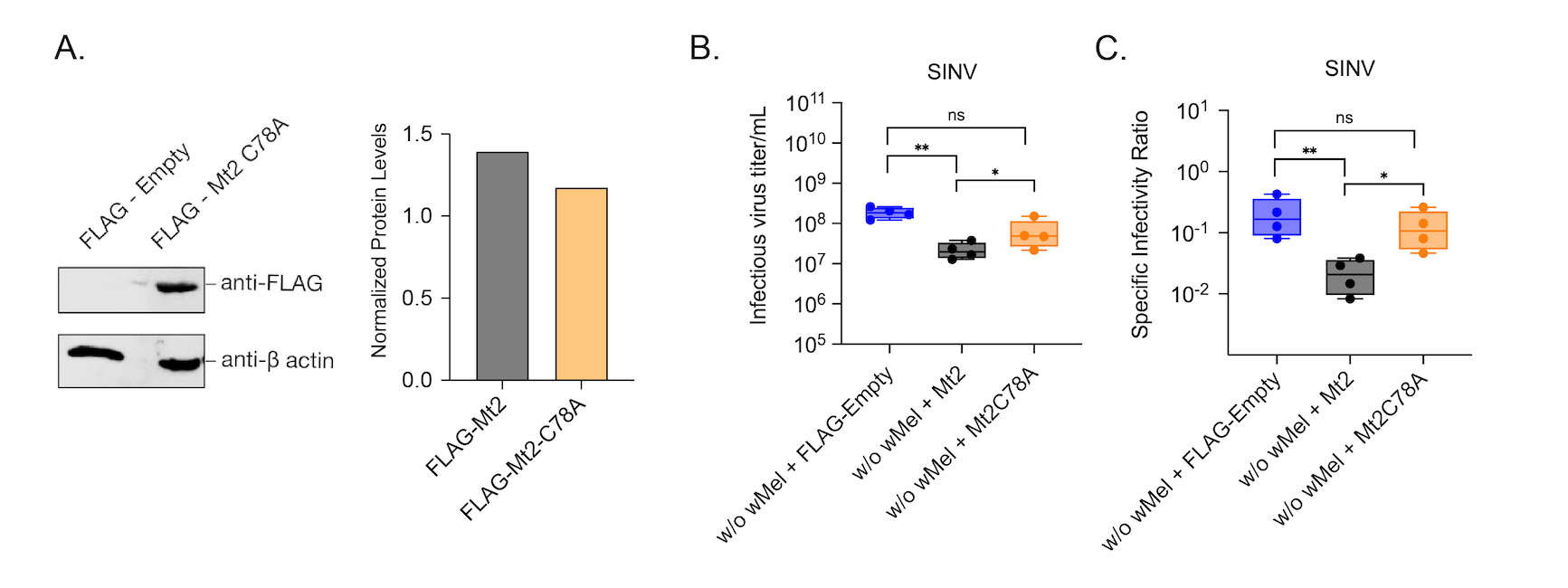

Supplement: S5 Fig — (A) Expression of the catalytic mutant of fly DNMT2 in Wolbachia-free D. melanogaster JW18 cells was assessed by Western Blot 72 hours post transfection with either the epitope tagged Mt2 mutant (FLAG-Mt2 C78A) or the empty control vector (FLAG-Empty) plasmid. Bar graphs represent wild-type (S4A Fig) and C78A Mt2 mutant protein levels normalized to β-actin controls. (B) 72 hours after Wolbachia-free D. melanogaster JW18 cells were transfected with plasmids carrying either the wild-type (FLAG-Mt2), catalytic mutant (FLAG-Mt2 C78A) or the empty control vector (FLAG-Empty), they were challenged with SINV at MOI of 10. Cell supernatants were harvested 48 hours post infection, clarified, and used to assess Infectious SINV titer by standard plaque assay on vertebrate BHK-21 cells. One-way ANOVA with Tukey’s post hoc test for multivariate comparisons. Error bars represent the standard error of mean of independent experimental replicates. (C) Specific Infectivity Ratios of progeny viruses produced 48 hours post infection was measured as the ratio of infectious virus titer (presented in B) to viral genome copies present in the cell supernatant, quantified using qRT-PCR using primers probing SINV E1 gene (see Materials and Methods for more details on the procedure and S1 Table for primer details). One-way ANOVA with Tukey’s post hoc test for multivariate comparisons. Error bars represent the standard error of mean of independent experimental replicates. *P < 0.05, **P < 0.01, ns = non-significant. (TIF) [file ppat.1010393.s005.tif]

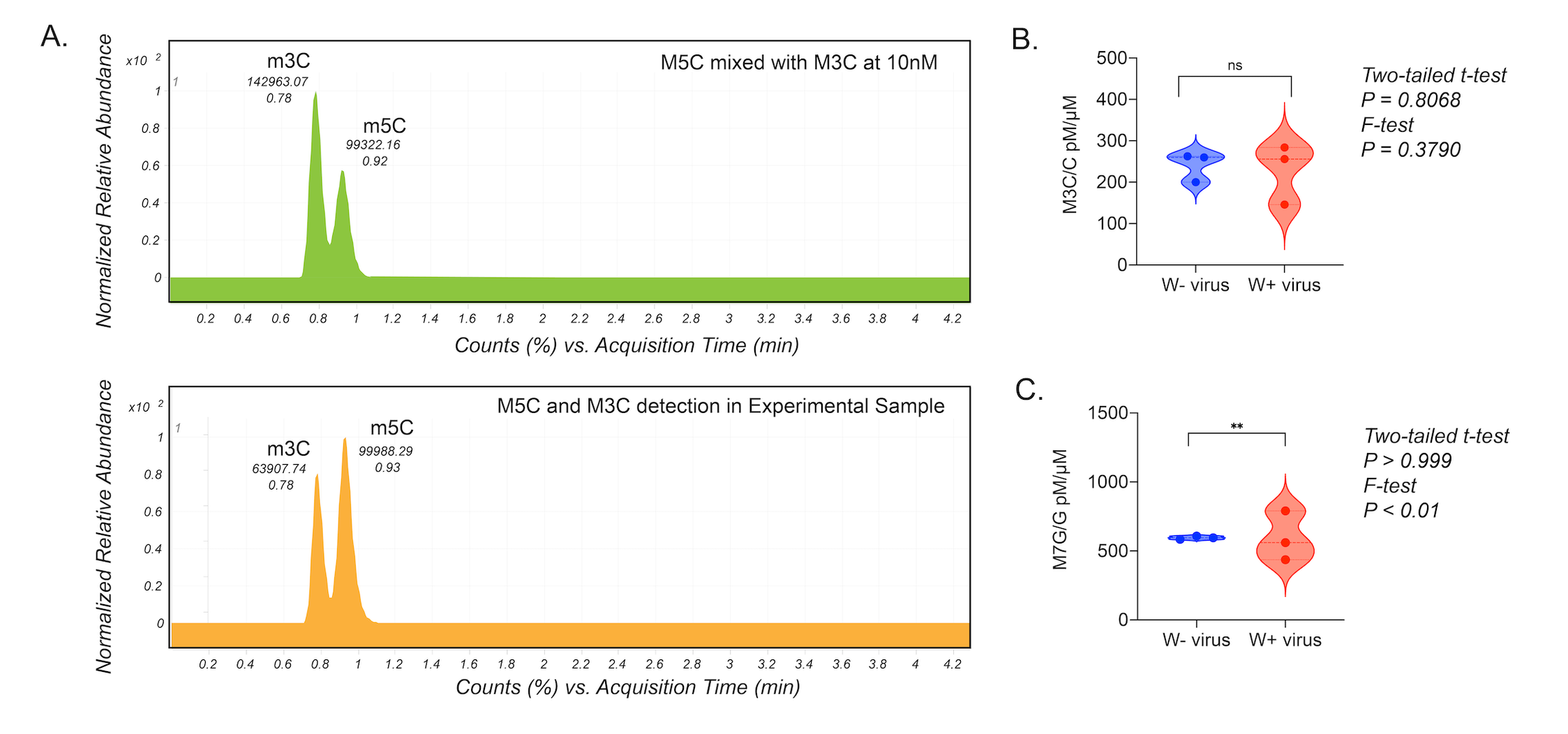

Supplement: S6 Fig — (A) Chromatograms representing simultaneous detection of 3-methyl cytosine (m3C) and 5-methyl cytosine (m5C) groups in mixture comprised of 10nM of each standard (Top) and one representative experimental sample (Bottom). (B) Normalized 3-methyl cytosine content of RNA isolated from W- and W+ viruses represented as a ratio of total unmodified cytosine content. Unpaired two-tailed t-test, p = 0.8068, t = 0.2612, df = 4 (C) Normalized 7-methyl guanosine content of RNA isolated from W- and W+ viruses represented as a ratio of total unmodified guanosine content. Unpaired t-test with Welch’s correction and F-test to compare variances. Error bars represent standard error of mean (SEM) of three independent virus preps from each cell type. F-test results: **P < 0.01, ns = non-significant. (TIF) [file ppat.1010393.s006.tif]
