# Supplementary material for: Differential viral RNA methylation contributes to pathogen blocking in Wolbachia-colonized arthropods
Source: PLoS Pathog. 2022 Mar 16;18(3):e1010393. doi: 10.1371/journal.ppat.1010393 (PMC8959158; doi:10.1371/journal.ppat.1010393)
Supplement: S1 Table — Primers were purchased from Integrated DNA Technologies (IDT). All primers were used at a final concentration of 10μM for quantitative PCR and RT-PCR reactions. Recommended primer concentrations according to manufacturer’s protocol were used for cloning experiments. (DOCX) [file ppat.1010393.s007.docx]

| **Primer Name** | **Forward Primer Sequence (5’-3’)** | **Reverse Primer Sequence (5’-3’)** |
| --- | --- | --- |
| CHIKV E2 | GGAATAAAGACGGATGATAGC | GGTCGGGAATGAAATTTTTCC |
| SINV E1 | TCAGATGCACCACTGGTCTCAACA | ATTGACCTTCGCGGTCGGATACAT |
| SINV nsP1 | AAGGATCTCCGGACCGTACTTG | CATGAACTGGGTGGTGTCGAAGC |
| Aedes 18S | CGAAAGTTAGAGGTTCGAAGGCGA | CCGTGTTGAGTCAAATTAAGCCGC |
| WSP | CATTGGTGTTGGTGTTGGTG | ACCGAAATAACGAGCTCCAG |
| Aedes GAPDH | CCGCTGATCTGCTAAACATAGA | GTTCTTCCGGGAGGATTCATTAG |
| Fly Mt2 | CCGTGGCGTGAAATAGCG | ACACCGCTTTCGGAGGACG |
| Aedes AMt2 | TATCAATCCGGTGGCCAATAC | CGGCGGTGACATGAGAATAA |
| pAFW-Mt2 QC_SaII | ACAAGGATGACGATGACAAGGTCCGAC | GGGTCGGCGCGCCCACCCTTGTCGAC |
| pAFW-Mt2_GA_Insert | AGGATGACGATGACAAGGTCATGGTATTTCGGGTCTTAGA | TCGGCGCGCCCACCCTTGTCTCATTTTATCGTCAGCAATT |
| pAFW-AMt2 | GCAACCGGTTTATGAGTGTTACCGACGGA | GCAGCTAGCTCAGTCCATCTCATCAAACAACGAACTC |
| Mt2-C78A_QC | GTCCCCGCCAGCTCAGCCCCACAC | ATCAGCAGCATGTTGGCC |
| AMt2-C78G_QC | GTCACCGGGCCAACCATTCA | ATGAGAGTAACGTTCACACCAAGCTTCTGAATG |
